# NanoSIMS sulfur isotopic analysis at 100 nm scale by imaging technique

**DOI:** 10.3389/fchem.2023.1120092

**Published:** 2023-03-16

**Authors:** Jia-Long Hao, Liu-Ping Zhang, Wei Yang, Zhao-Yang Li, Rui-Ying Li, Sen Hu, Yang-Ting Lin

**Affiliations:** ^1^ Key Laboratory of Earth and Planetary Physics, Institute of Geology and Geophysics, Chinese Academy of Sciences, Beijing, China; ^2^ Key Laboratory of Petroleum Resource, Institute of Geology and Geophysics, Chinese Academy of Science, Beijing, China; ^3^ Institute of Disaster Prevention, Sanhe, China

**Keywords:** NanoSIMS, sulfur isotope analysis, isotopic images, spatial resolution, analytical precision

## Abstract

NanoSIMS has been widely used for *in-situ* sulfur isotopic analysis (^32^S and ^34^S) of micron-sized grains or complex zoning in sulfide in terrestrial and extraterrestrial samples. However, the conventional spot mode analysis is restricted by depth effects at the spatial resolution < 0.5–1 μm. Thus sufficient signal amount cannot be achieved due to limited analytical depths, resulting in low analytical precision (1.5‰). Here we report a new method that simultaneously improves spatial resolution and precision of sulfur isotopic analysis based on the NanoSIMS imaging mode. This method uses a long acquisition time (e.g., 3 h) for each analytical area to obtain sufficient signal amount, rastered with the Cs^+^ primary beam of ∼100 nm in diameter. Due to the high acquisition time, primary ion beam (FCP) intensity drifting and quasi-simultaneous arrival (QSA) significantly affects the sulfur isotopic measurement of secondary ion images. Therefore, the interpolation correction was used to eliminate the effect of FCP intensity variation, and the coefficients for the QSA correction were determined with sulfide isotopic standards. Then, the sulfur isotopic composition was acquired by the segmentation and calculation of the calibrated isotopic images. The optimal spatial resolution of ∼ 100 nm (Sampling volume of 5 nm × 1.5 μm^2^) for sulfur isotopic analysis can be implemented with an analytical precision of ∼1‰ (1SD). Our study demonstrates that imaging analysis is superior to spot-mode analysis in irregular analytical areas where relatively high spatial resolution and precision are required and may be widely applied to other isotopic analyses.

## 1 Introduction

Sulfur isotopes have been used as an important tracer to study igneous, metamorphic, sedimentary, hydrothermal, and biologic processes (Farquhar et al., 2000; Seal, 2006; Marini et al., 2011; Wang et al., 2021). Secondary ion mass spectrometry (SIMS) has been widely applied to *in situ* sulfur isotopic analysis with a high spatial resolution (Winterholler et al., 2008; Kozdon et al., 2010; Nishizawa et al., 2010; Wynn et al., 2010; Kita et al., 2011; Whitehouse, 2013; Deditius et al., 2014; Ireland et al., 2014; Ushikubo et al., 2014; Zhang et al., 2014; Hauri et al., 2016; Jones et al., 2018; Liu et al., 2021). For example, Cameca NanoSIMS 50 can perform sulfur isotope analysis down to the micron-submicron scale, which has become a valuable tool for studying fine-grained sulfides or those with complex zonings (Zhang et al., 2017; Liang et al., 2019; Gao et al., 2022). A sulfur isotope analytical method of individual micron-sized aerosol particles was established to identify their sources (Winterholler et al., 2008). *In-situ* sulfur measurements of micron-size framboid pyrite have provided a record of the isotope fractionation from the early Ediacaran ocean sulfate (Hu et al., 2021; Wang et al., 2021). Recently, micron-scale resolution of the NanoSIMS analysis of S isotopes has been conducted on sulfides from the CE-5 samples to constrain the S abundance and isotopic composition of the CE-5 mantle source, which is the first report of S isotope compositions of magmatic sulfides from lunar mare basalts (Liu et al., 2022). In these previous studies, the analytical spatial resolution of 2–5 microns was usually used to perform S isotope analysis with the analytical precision 0.3‰–1‰ (1SD) (Zhang et al., 2014; Hauri et al., 2016). However, sub-micron scale to nanoscale chemical heterogeneities in pyrite have been observed (Deditius et al., 2014; Li et al., 2018; Yan et al., 2018; Gopon et al., 2019; Holley et al., 2019; Wu et al., 2019). The analytical accuracy becomes significantly worse when performing *in-situ* S isotope analysis for these samples with a sub-micron scale. Using the spot analysis mode of the NanoSIMS 50L, a reproducibility of 1.5‰ (1SD) for ^34^S/^32^S ratios with a lateral resolution of ∼ 0.5 μm has been reported (Zhang et al., 2014). Thus, such spatial resolution and precision are insufficient to decipher the complex hydrothermal processes and growth kinetics of the pyrite. An *in-situ* sulfur isotopic method with higher spatial resolution and precision is still required for determining the sulfur isotope of submicron-scale to nanoscale oscillatory zoning or complex core-rim structure in pyrite.

Although the smallest probe size for NanoSIMS can be set to < 50 nm, the spatial resolution for sulfur isotopic analyses (spot size + raster size) is considerably larger than 50 nm. The main reason is that when small probe sizes are used to achieve a high spatial resolution, the ion current of the primary beam and the intensity of the secondary beam are both reduced. Generally, the acquisition time is lengthened to obtain a sufficient signal for statistical purposes. However, rastering on a small area with a long acquisition time can cause noticeable depth effects, which result in changes in the angle at which the second ion is sputtering. The Instrumental Mass Fractionation (IMF) will change accordingly, resulting in a decrease in analytical accuracy (Yang et al., 2012; Farquhar et al., 2013; Hao et al., 2021). To reduce the impact of depth effect on analytical accuracy, different IMF factors was calculated and applied in each analysis by Farquhar et al. (2013) with a static beam of 10 μm using a Camecca IMS1280. Generally, in spot mode of NanoSIMS at the spatial resolution of micron to sub-micron scale, a larger raster area was set to eliminate the depth effect on IMF, which, however, leads to a worse spatial resolution. Thus, in SIMS spot analysis, high spatial resolution (< 1 μm) cannot be achieved equal to the size of the primary beam.

An important function of NanoSIMS is its ion image mode. This mode is primarily used to observe the isotopic or elemental distributions using small probe sizes (down to 100 nm). The secondary ion image is acquired by rastering the primary beam over a larger area, thereby eliminating the crater effect. The isotopic composition can be determined by segmenting and calculating the secondary ion images. We report here a new method based on the NanoSIMS imaging mode that improves both spatial resolution and precision of sulfur isotopic analyses. The optimal spatial resolution of ∼ 100 nm (Sampling volume of 5 nm × 1.5 μm^2^) for S isotopic analysis can be implemented with an analytical precision of ∼1‰ (1SD). This method is particularly suitable for sulfur isotopic analysis of submicron-scale to nanoscale oscillatory zoning or complex core-rim structure.

## 2 Methods

### 2.1 Material description

The measured (^34^S/^32^S) ratios are reported as δ^34^S in the standard per mil notation (‰):

$$\delta^{34}S=\left[({\;}^{34}S/{\;}^{32}S_{sample})\;/\;{\;}^{34}S/{\;}^{32}(S_{standard})-1\right]\times 1000$$ (1)

where the standard adopted is VCDT (Vienna Canyon Diablo Troilite) with a ^34^S/^32^S value of 044162. Three natural pyrite standard samples have been used in our method, including PY-CS01 (δ^34^S = 4.6‰ ± 0.1‰ 1SD), PY 1117 (δ^34^S = 0.3‰ ± 0.1‰)and PY-SRZK (δ^34^S = 3.6‰ ± 0.05‰). Pyrite standard Py-1117 was used as the reference material, and the sulfur isotopic measurement was conducted on the standards of PY-CS01 and PY-SRZK used as the test samples. The bulk sulfur isotopic compositions of the pure mineral separations were determined by a conventional method (gas-source mass spectrometry). All the standards were embedded in epoxy resin and prepared as a polished disk with a diameter of 1 inch. Then coated with carbon and mounted in the sample holder.

### 2.2 Instrumental settings

**FIGURE 1:**
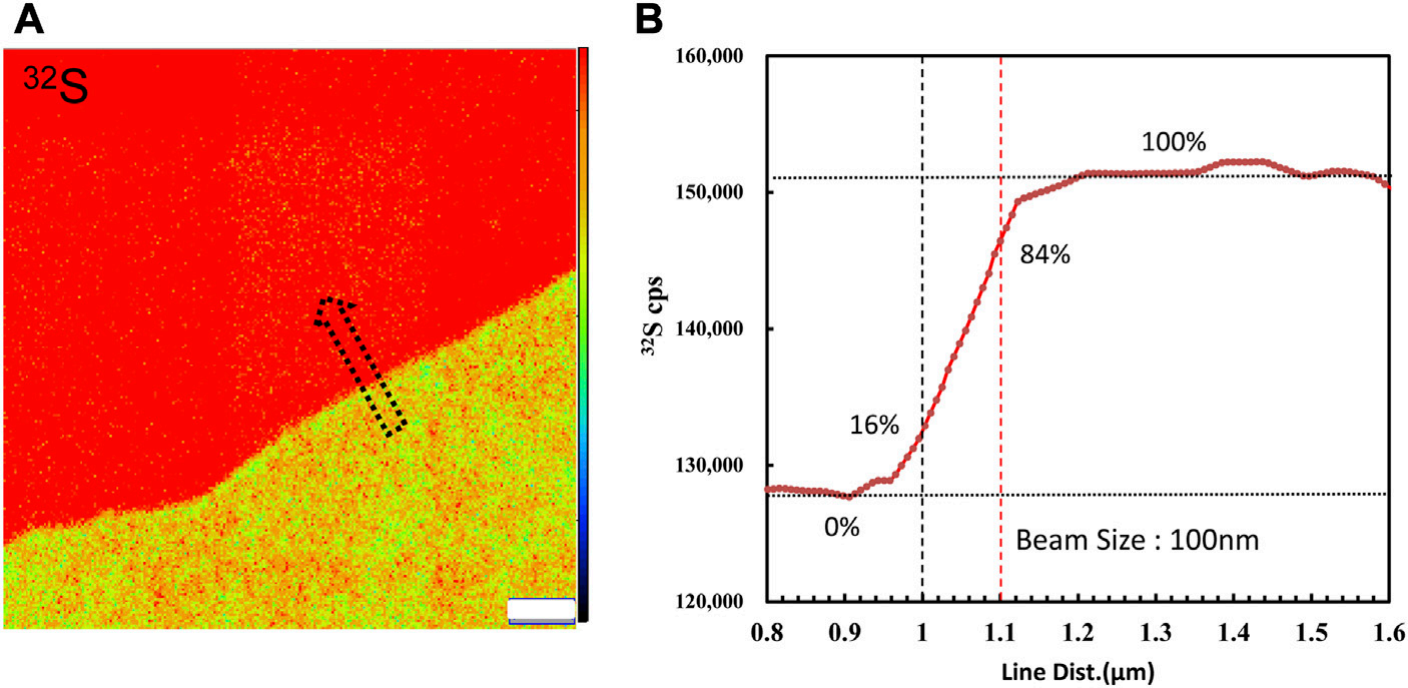
**(A)** ^32^S ion image acquired by raster a Cs bean of∼1 pA on the boundary of the coexistence of pyrite and chalcopyrite. **(B)** Profile of ^32^S CPS along the arrow. The primary beam size was measured to be 100 nm by the knife-edge method. The width estimation error is 0.3%.

The *in-situ* isotopic measurements of sulfur were performed in the NanoSIMS Lab at the Institute of Geology and Geophysics, Chinese Academy of Sciences (IGGCAS). Ions image mode is used to acquire the ions image of ^32^S and ^34^S by raster the primary beam counting with electron multiplier (EMs). To optimize the primary beam setting with the spatial resolution and the signal amount for statistics, the Cs + beam of ∼ 1 pA and a diameter of∼ 100 nm was used (Figure 1). In this condition, the count rates of ^32^S and ^34^S are ∼3–4 × 10^5^ and ∼1.2–1.6 × 10^4^ counts per second (CPS), respectively. These counting conditions can meet the needs of a sufficient signal amount of ^34^S and reduce the EM aging effect of ^32^S. Details on the aging effect and the signal amount for statistics are discussed in later sections. In order to eliminate interference from ^32^SH_2_^−^, ^33^SH_1_^−^ on 34S^−^, the entrance slit (30 μm), apertures slit (200 μm) and exit silt (90 μm) were used to achieve a mass resolution of ∼7,000. Multi EM detectors were set to acquire the ions image of ^32^S, ^34^S, ^60^Ni, ^80^Se, ^63^Cu^32^S, and ^75^As^32^S. Among them, ^60^Ni-, ^80^Se-and ^75^As^32^S-are zoning related elements. ^63^Cu^32^S is used to eliminate some areas where chalcopyrite and pyrite are accompanied. In addition, the secondary electrons are also detected to represent the surface appearance characteristics. The NMR Probe is used to stabilize the magnetic field during the multi-collection.

### 2.3 Ions image acquisition and data processing

The scanning imaging size of each area on the samples is 20 × 20 μm^2^. Before collecting the ions image, each area was presputted with a primary beam of ∼1 nA for∼ 30 s to 1 min to remove the carbon film on the surface and implant ions to obtain a stable yield. To compensate for the changes of sample height of different analytical location, EOS centering and SIB (secondary ion beam) centering were applied before each measurement. It is necessary to clarify that if the standard sample and unknown sample are in different holder, the EOS voltage are required to be adjusted to be consistent by changing the Z-axis. In addition, automatic peak centering was employed before acquiring the ions images. Under the primary beam condition of ∼1 pA with 20 × 20 μm^2^ raster size, the total counting time takes at least 2 h to acquire the analysis precession of 1‰ at the spatial resolution of 100 nm (discussed in later sections). The count rate of ^32^S is ∼ 300000–400000 cps. In order to reduce the impact of image drift, each area has been raster in 30 frames, with 256 × 256 pixels and 5 ms/pixel dwell time. The total duration is 9,900 s for each area and the sputter depth is ∼5 nm calculated using the sputter rate (0.2 nm*μm^2^ pA−1 s−1) report in the previous study (Xu et al., 2022). The acquired ion images were processed and analyzed using ImageJ with the Open MIMS plugin. The 30 frames of ion images of each isotope were automatically aligned and added using the TurboReg ImageJ plugin (Hao et al., 2020). Then the Region of Interesting (ROI)s were selected depended the Experimental requirements. The total counts of ^32^S and ^34^S in the ROI area was calculated and output from Image J as the raw data of the isotope for processed. The raw data were first corrected for dead time effect (Supplementary Note S1). After that, the measured ^34^S/^32^S were corrected for QSA and Matrix effect, which is described in later section.

## 3 Factors affecting S isotopic measurement precision using ions image mode

### 3.1 Ions image acquisition time and aging effect of the electron multiplier

Using NanoSIMS ions image mode, the secondary ions images can be used to calculate the elemental content or isotopic ratio of the region (ROI). According to Poisson’s statistical theory, the analytical precision of ^34^S/^32^S measurements depend on total signal statistics. Indeed, the total signal statics of ions image mode depend on the primary ions beam current and the acquisition time. We have calculated that at least more than 10,000 s of the acquisition time is required to obtain an analytical precision of 1‰ at the spatial resolution of ∼100 nm. The detailed information is described in the (Supplementary Note S2).

**FIGURE 2:**
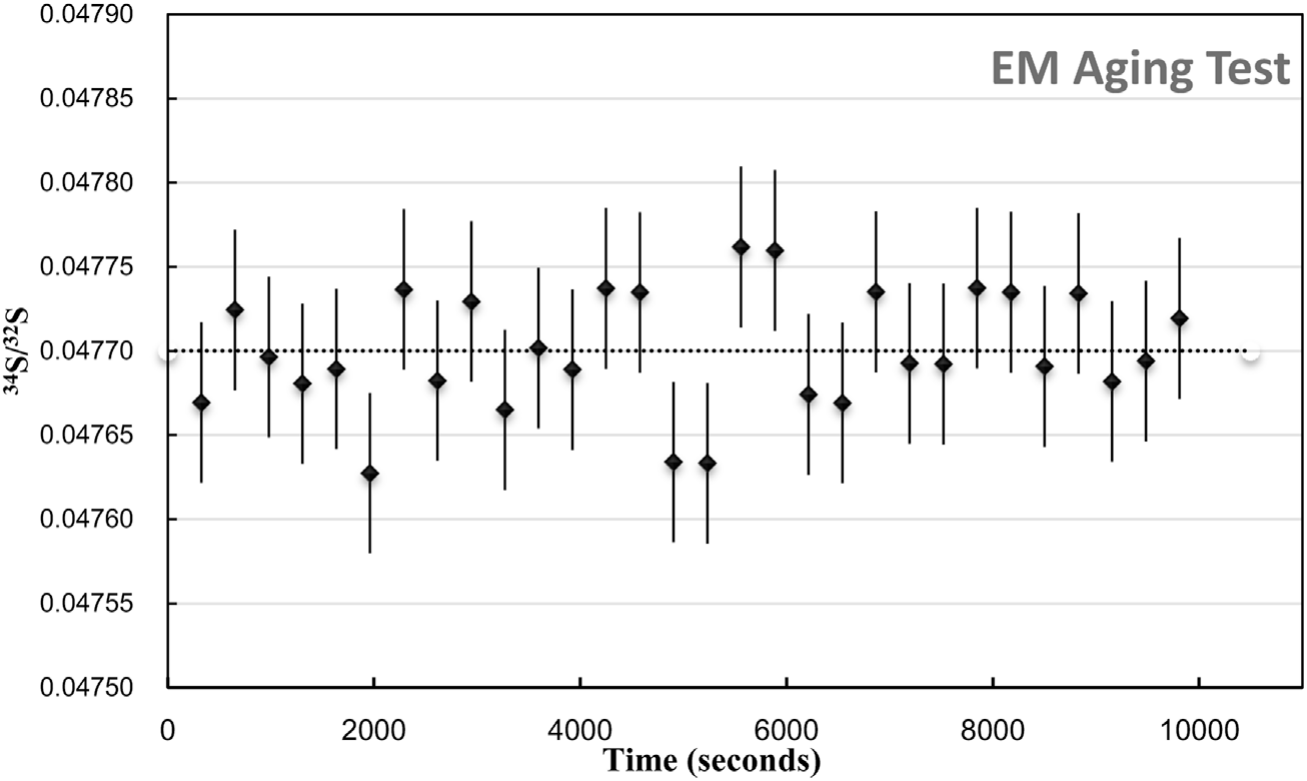
EM aging test. The plot of ^34^S/^32^S of 30 Frame within 9,900 s duration. The err bar is 1SE.

Compared with the several minutes of analysis time used in spot mode, the longer detected time of each analysis was employed with electron multiplier. The aging effect of the EM is required to be considered. The aging effect of EM will occur when used to receive higher intensity secondary ions (more than 400000 cps), lowering the gain of the electron multipliers (the number of secondary electrons per secondary ions). It causes the isotope ratio drift of more than 1‰–2‰ per hour. In order to eliminate the aging effects on the isotopic analytical precision, two methods are mainly used for correction: 1) after analyzing ∼10 unknown samples, measure the references standards and correct the ratio. It is more commonly used and suitable for spot analysis with a shorter analysis time for each measurement. 2) Increasing the high voltage of EMs, the peak height distribution (PHD)_MAX_ is located in the 240–290 mv interval, which can eliminate aging effects to obtain a high precision (0.5‰) isotope analysis. These two methods are combined in our study. The standards are used to monitor the drift, and the PHD_MAX_ of EMs is maintained within a required range. We have tested the age effect on each analytical section. Each area has been raster in 30 frames, with 256 × 256 pixels and 5 ms/pixel dwell time. The total duration is 9,900 s for each area. As shown in Figure 2, during the analysis, the S isotopic ratio between each layer does not have a significant change trend, and the precision of the isotope between each layer is 0.74‰ (1RSD relative standard deviation).

### 3.2 QSA effect in imaging mode

Quasi-simultaneous arrivals (QSA) effect may induce a specific type of bias. It is a challenge in high-precision analysis using EMs. For the QSA effect correction of S isotope analysis, Slodzian calculated Kcor (the average number of secondary ions ejected per primary ion) with an iterative procedure through probability distribution (Slodzian et al., 2001; Slodzian et al., 2004):

$$Kcor=Kexp\;/\;\left(1-0.5\times Kexp\right)$$ (2)

Where Kexp is the experimental ratio of secondary intensity over primary intensity. Then the QSA coefficient from the linear correlation between the measured isotope ratios has been determined:

$$\delta^{34}S_{cor}=\delta^{34}S_{\exp}-\beta \times Kcor\times 1000$$ (3)

**FIGURE 3:**
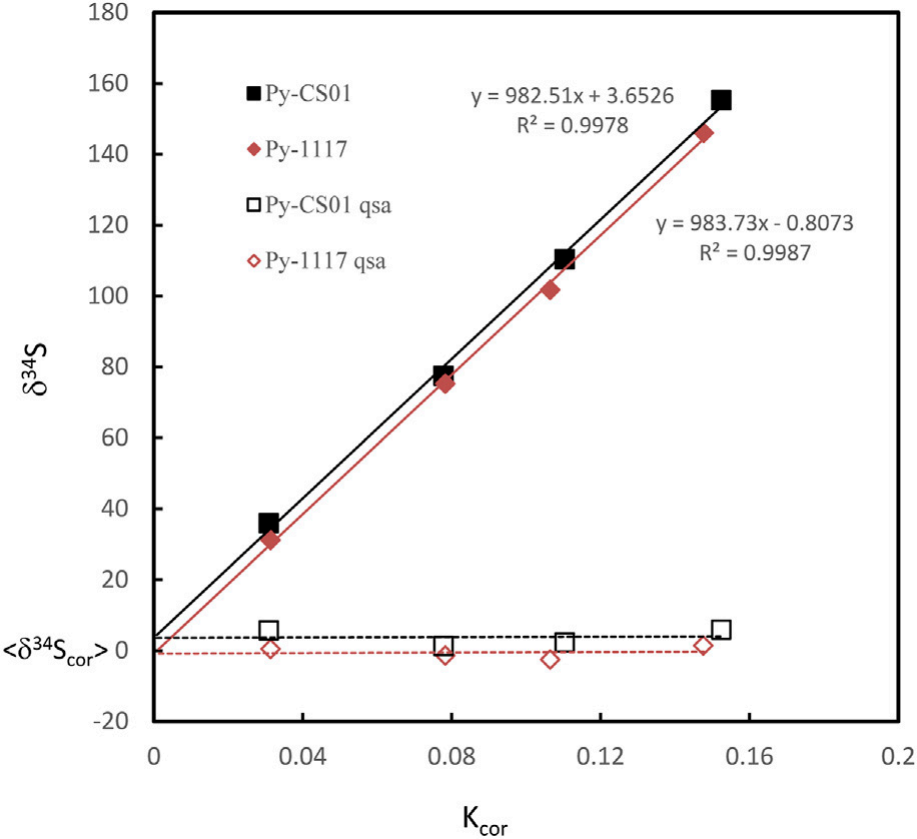
The plot of the relation between δ34S and Kcor before and after QSA correction. The QSA coefficient β (slope) of ∼ 0.98 is determined. The slope is consistent within the fitting error in these two pyrite standards on different S isotopic compositions. The gap between the fitting line intercept indicates the difference in the compositions of the S isotope.

Where β is the QSA coefficient. QSA correction and the QSA coefficient β in spot analysis of SIMS have been determined by various types of ion probes in previous studies. However, there is no report of the sulfur isotope QSA correction in ion image mode. Therefore, we carried out S isotopic QSA correction using ions image mode on two known pyrite standards, Py-1117 (δ^34^S = 0.3‰ ± 0.1‰) and Py-CS01(δ^34^S = 4.6‰ ± 0.1‰). In order to determine the coefficient β of various K values, different widths of aperture slits (AS1, 2, 3, and 4) have been used. The ion image acquisition time of each condition varies from 10 to 30 min, depending on the ion intensity. The sulfur isotopic data of QSA correction and K values measured using various aperture slit settings are given in Table 1. The plot of the relation between δ^34^S and Kcor before and after QSA correction is shown in Figure 3. It can be seen that δ^34^Sexp exhibits a linear relationship to Kcor, which is related to the QSA effect. The QSA coefficient β (slope) of ∼ 0.98 is determined in our method. The slope is consistent in these two pyrite standards with different S isotopic compositions. The gap between the fitting line intercept indicates the difference in the compositions of the S isotope. Therefore, the QSA correction formula is determined:

$$\delta^{34}S_{cor}=\delta^{34}S_{\exp}\;–\;0.98\times Kcor\times 1000$$ (4)

**TABLE 1:** The sulfur isotopic data of QSA correction and the K values measured using various aperture slits on the standards of PY-1117 and PY-CS01.

Sample	AS	K_exp_	K_cor_	δ^34^S_exp_	δ^34^S_cor_
PY-1117	1	0.137	0.148	146.1	0.9
PY-1117	2	0.101	0.106	101.8	−2.8
PY-1117	3	0.075	0.078	75.3	−1.7
PY-1117	4	0.031	0.031	31.2	0.4
PY-CS 01	1	0.142	0.152	155.3	5.6
PY-CS 01	2	0.104	0.110	110.4	2.1
PY-CS 01	3	0.075	0.078	77.5	1.2
PY-CS 01	4	0.030	0.031	35.9	5.7

**FIGURE 4:**
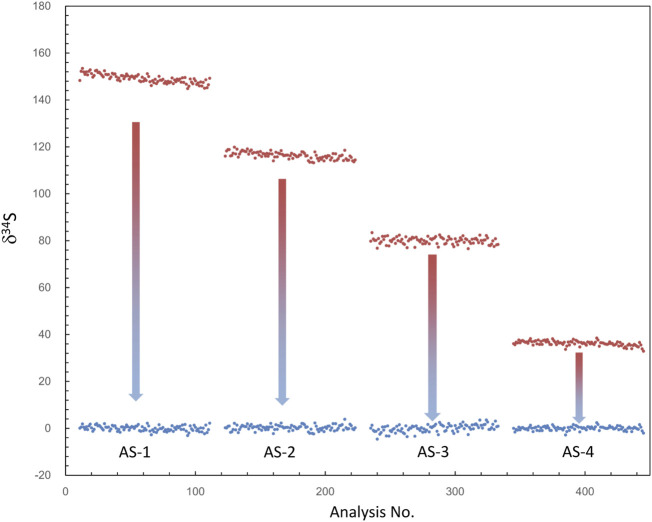
The plot of δ^34^S of each frame measured using different aperture slits before and after QSA correction. δ^34^Sexp (before the correction) is shown in the red plot, and δ^34^Scor (after the correction) is shown in the blue plot. δ^34^Sexp presented a trend of less instrumental mass fractionation with the decrease of aperture slit width, leading to the decrease of the K value.

Note that if the instrument switches to the other analysis method and returns to the S isotope measurement, the β value and the calibration curve must be re-established. In addition, as shown in Table 1, δ^34^Scor measured using different silts is not shown as consistent. The instrumental mass fractionation (IMF) may vary with the used AS width, and the mass resolution is inconsistent. Therefore, IMF correction of the corresponding instrument settings, in which AS1 is used in this method, should be carried out after QSA correction. Moreover, the plot of raw δ^34^S of each frame on PY-1117 was measured using different aperture slits before and after QSA correction is given in Figure 4. δ^34^Sexp presented a trend of less instrumental mass fractionation with the aperture slit width decreasing. Indeed, with the decrease of the K value, the influence of the QSA effect gradually becomes smaller.

**FIGURE 5:**
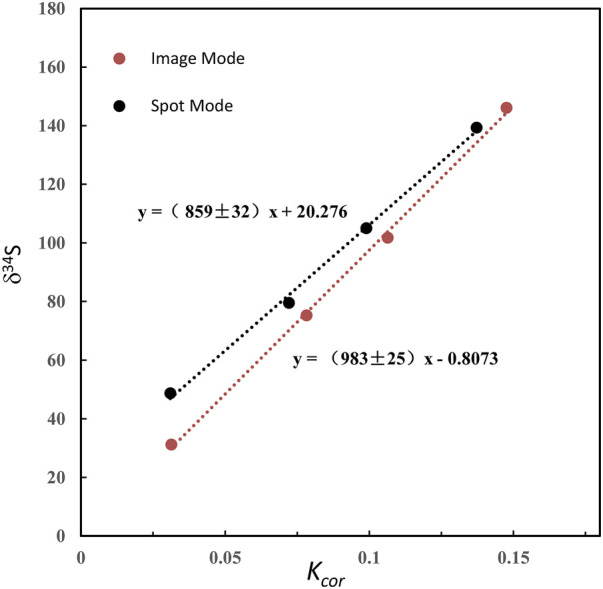
The plot of the relation between δ^34^S and Kcor in image mode and spot mode.

Compared with the QSA coefficient β measured by previous studies in the range of 0.7–0.8, the value determined in our experiment is relatively higher. It is maybe due to the IMF difference between QSA correction in spot analysis mode and QSA correction in image analysis mode. QSA in spot analysis mode was also tested in our experiment. The raster size was decreased to 2 × 2 μm^2^, with other instrumental settings remaining the same. As shown in Figure 5, the slope of spot mode is ∼0.86, significantly lower than that of image mode (∼0.98), which is close to spot mode in previous studies.

### 3.3 Primary beam current stability

The stability of the primary beam current of the NanoSIMS 50L is less than 1% in 10 min. In the conventional spot analysis mode, the stability of the primary beam is not considered. For most isotope analyses, the impact of the instability of the primary beam is much lower than the analysis accuracy requirements. Even when analyzing the high-precision isotope in spot mode with Electronic Multipliers, due to the short analytical time, the impact of the changes in primary beam current is much lower than the analysis precision. Furthermore, the influence can be further reduced by calculating the K value of the primary beam intensity measured before each analysis. However, high-precision isotope image analysis requires 2–3 h or an even longer time, and the stability of the primary beam current may affect the accuracy of the isotope results. In our experiment, the influence of primary beam intensity variation on QSA correction was observed. The change of Cs^+^ primary ion beam intensity is mainly caused by the variation of the evaporation of Cs₂CO_3_ in the ion source and the change of ionization of Cs+, which is mostly monotonic change within a few hours. Consequently, the primary ion beam intensity, which is called *FCP* (Faraday Cup Primary) on the NanoSIMS, was measured before (*FCP*_*before*_) and after (*FCP*_*after*_) the multilayer isotope image analysis. Then the *FCP*_*before*_ and the *FCP*_*after*_ were interpolated into each layer of images to determine the *FCP*_*(i)*_ of frame *i* (Formula 5).

$${FCP}_{cor\left(i\right)}={FCP}_{before}+\frac{\left({FCP}_{after}-{FCP}_{before}\right)}{N}\times i$$ (5)

**FIGURE 6:**
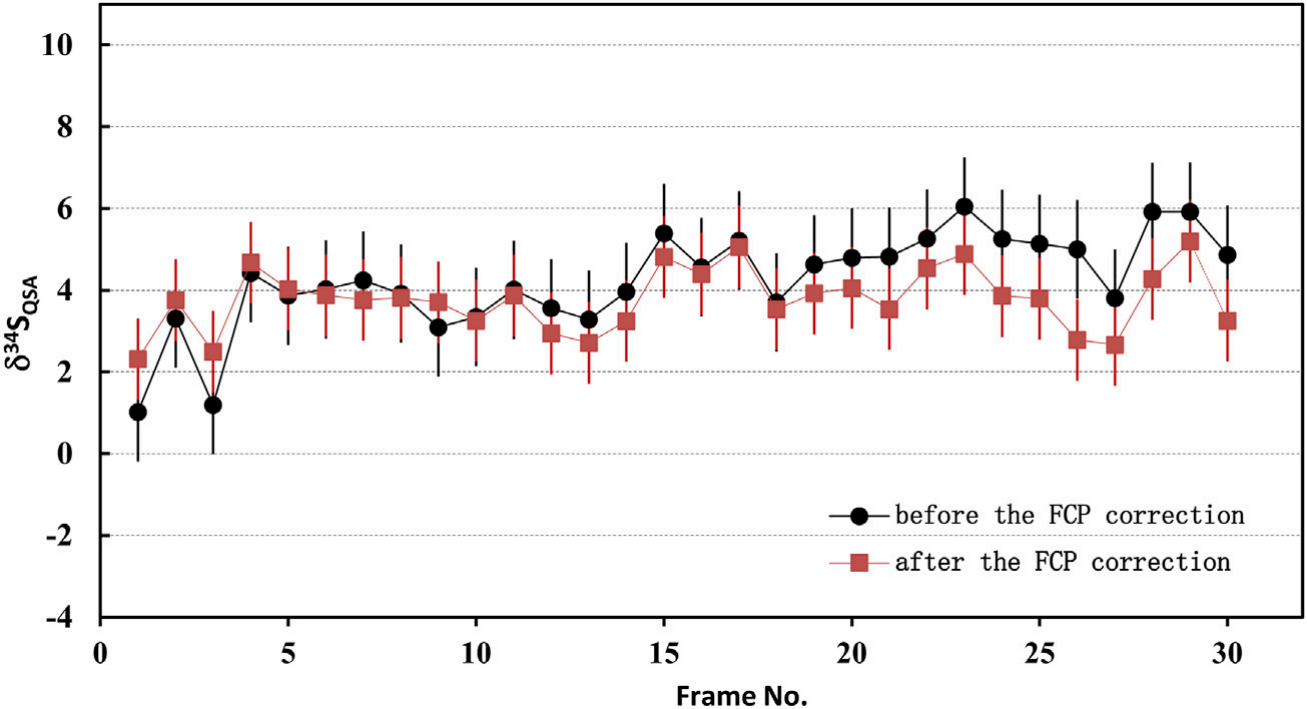
The plot of the S isotopic results before and after FCP correction measured on PY-SRZK pyrite standard.

Where *N* is the total frame number of ions image, *FCP*_*cor(i)*_ is the *FCP* of the frame *i* after correction. Accordingly, the primary ion beam bombarded on the sample of each frame, which was used to calculate the K value, was determined by the relationship between the primary ion beam FCP_(*i*)_ and the bombarded ion beam on the sample surface (*FCO* in the NanoSIMS). Then, the QSA correction was performed using the corresponding K value and β (Formula 3). To illustrate the impact of the FCP correction, the S isotopic data before and after FCP correction measured on PY-SRZK pyrite standard are shown in Table 2 and plot in Figure 6. The two sets of data are given respectively: 1) The average value of primary beam current before (FCP_before_) and after (FCP_after_) in the ions image acquisition, is applied to calculation the K value. The δ^34^S values were calculated after correction of the QSA effect using this K value; 2) The interpolated FCP using our method (Formula 5) was applied to determine the K value and correct the QSA effect. As shown in Table 2, the primary beam current measured before and after the ions image acquisition were 25670 pA and 25300 pA, respectively, indicating that the beam intensity decreased by 1.4% during the measurement of about 3 h. For the first set of data, there was no primary beam FCP correction, and the ^34^S_qsa_ showed a noticeable monotony change (Figure 6). The decrease of the primary beam caused an inaccurate calculation of Kcor. However, the second set of data after the FCP correction shows no trend related to time/frame, and the δ^34^S_qsa_ are relatively consistent. Accordingly, the analytical precision has been improved from 1.2‰ (1 RSD) to 0.8‰. Hence, it is necessary to perform primary beam current (*FCP*) correction on the results of each frame in analyzing high-precision stable isotopes in image mode. Then, the QSA correction can be performed to obtain more precise results.

**TABLE 2:** The S isotopic results before and after *FCP* correction measured on the pyrite standard PY-SRZK.

*Frame*	^*34*^ *S/* ^*32*^ *S*	*δ* ^*34*^ *S*_*Raw*_	*FCP*_*AVE.*_ *(pA)*	*K*_*cor*_ *^a^*	*δ* ^*34*^ *S*_*QSA*_ *^a^*	*FCP*_*COR.*_ *(pA)*	*K*_*cor*_ *^b^*	*δ* ^*34*^ *S*_*QSA*_ *^b^*
1	0.050767	149.6	25,485	0.150	1.6	25,670	0.149	2.9
2	0.050841	151.2	25,485	0.150	3.9	25,657	0.149	4.3
3	0.050738	148.9	25,485	0.150	1.8	25,644	0.148	3.1
4	0.050869	151.8	25,485	0.149	5.0	25,632	0.148	5.2
5	0.050837	151.1	25,485	0.149	4.4	25,619	0.148	4.6
6	0.050838	151.1	25,485	0.149	4.6	25,606	0.148	4.4
7	0.050839	151.2	25,485	0.149	4.8	25,593	0.148	4.3
8	0.050825	150.9	25,485	0.149	4.5	25,581	0.148	4.4
9	0.050779	149.8	25,485	0.149	3.6	25,568	0.148	4.3
10	0.050786	150.0	25,485	0.148	3.9	25,555	0.148	3.8
11	0.050812	150.6	25,485	0.148	4.6	25,542	0.148	4.4
12	0.050782	149.9	25,485	0.148	4.1	25,530	0.148	3.5
13	0.050763	149.5	25,485	0.148	3.8	25,517	0.148	3.3
14	0.050790	150.1	25,485	0.148	4.5	25,504	0.148	3.8
15	0.050844	151.3	25,485	0.148	6.0	25,491	0.147	5.4
16	0.050803	150.4	25,485	0.148	5.1	25,479	0.147	5.0
17	0.050826	150.9	25,485	0.148	5.8	25,466	0.147	5.6
18	0.050755	149.3	25,485	0.147	4.3	25,453	0.147	4.1
19	0.050786	150.0	25,485	0.147	5.2	25,440	0.147	4.5
20	0.050790	150.1	25,485	0.147	5.4	25,428	0.147	4.6
21	0.050783	149.9	25,485	0.147	5.4	25,415	0.147	4.1
22	0.050797	150.2	25,485	0.147	5.8	25,402	0.147	5.1
23	0.050823	150.8	25,485	0.147	6.6	25,389	0.147	5.4
24	0.050783	149.9	25,485	0.146	5.8	25,377	0.147	4.4
25	0.050770	149.6	25,485	0.146	5.7	25,364	0.147	4.4
26	0.050757	149.3	25,485	0.146	5.6	25,351	0.147	3.3
27	0.050696	147.9	25,485	0.146	4.4	25,338	0.146	3.2
28	0.050786	150.0	25,485	0.146	6.5	25,326	0.146	4.8
29	0.050779	149.8	25,485	0.146	6.5	25,313	0.146	5.8
30	0.050726	148.6	25,485	0.146	5.4	25,300	0.146	3.8
*Averange*					4.8			4.3
*SD*					1.2			0.8

^a^ ***K***_***cor***_^***a***^: calculated using the average value of primary beam current before (FCP_before_) and after (FCP_after_) the ions image acquisition. ***δ*** ^***34***^ ***S***_***QSA***_^***a***^: the δ^34^S after the QSA, correction using the ***K***_***cor***_^***a***^.

^b^ *K*_*cor*_^*b*^: calculated using the *FCP*, correction in this method. ***δ*** ^**34**^ ***S***_***QSA***_^***b***^: the δ^34^S after the QSA, correction using the ***K***_***cor***_^***b***^.

### 3.4 Matrix effect

The matrix effect means instrumental mass fractionation varies with different matrixes. The standard–sample–standard bracket method was commonly used to calibrate the matrix effects. The IMF calibration should be performed using the same instrumental settings. In spot mode analysis, it was estimated by comparing the measured ^34^S/^32^S ratio with the reference values measured using gas source isotope ratio mass spectrometry (GS-IRMS). Similarly, the IMF calibration in image mode was determined by the standards:

$$IMF=\left(1000+\delta^{34}S_{measure}\right)/\left(1000+\delta^{34}S_{true}\right)$$ (6)

Where δ^34^S_measure_ and δ^34^S_true_ are the measured and recommended values of δ^34^S, respectively. Then, isotope image analysis was carried out on the sample to be tested, and IMF corrected the S isotope ratio to obtain the true value. In this study, the δ^34^S result of unknown sample was reported with associated 1SE uncertainty (err bar). And the uncertainty of the each ROI on the unknown sample (SE_sample_)is estimated as the square sum of the reproducibility of δ^34^S measurements on the corresponding reference pyrite of PY 1,117 (SD _standard_), the internal precision of each ROI on the sample (SE _ROI_) and the uncertainty of the reference values of the standards 1,117 (SD _ref._):

$${SE}_{sample}=\sqrt{{{SE}_{ROI}}^{2}+{{SD}_{standard}}^{2}+{{SD}_{ref.}}^{2}}$$ (7)

In summary, the sulfur isotopic analysis method based on the NanoSIMS imaging mode was established.

(1) Instrumental setting: According to the requirement of stable isotope image analysis, the instrument is set, including primary beam configuration, mass spectrometer configuration, and multi-collection configuration.
(2) The QSA correction coefficient: Isotope imaging was performed on the isotope standard samples with aperture slits of different sizes to obtain the QSA correction slope for isotope image analysis.
(3) The Instrumental Mass Fractionation (IMF) correction: On the sulfur isotopic standard sample, which matches the matrix of the sample to be tested, the instrumental mass fractionation is calculated according to the measured isotope ratio and the recommended value of the standard sample.
(4) Ions image acquisition on the unknown sample: acquire the S isotope ions image on the unknown samples with the same analytical conditions as those on the standard samples, including a raster size of 20 × 20 μm^2^, a total of 30 frames and each layer acquisition time of ∼300 s. The standard–sample–standard bracket method to calibrate the matrix effects and QSA effects. Specifically, the isotope imaging on the standard was performed before the ion image acquirement on each area of unknown sample.
(5) Data process of ion image on unknown sample: The 30 frames of ion images were automatically aligned and added. Then the Region of Interesting (ROI)s were selected depended the Experimental requirements. The total counts of ^32^S and ^34^S in the ROI area was calculated and output from Image J as the raw data of the isotope for processed. The raw data were first corrected for dead time effect. After that, the measured ^34^S/^32^S were corrected for FCP, QSA and Matrix effect.

## 4 Results and discussion

### 4.1 Internal precision of each ROI and Poisson error

Compared with the detected using Faraday Cup, the internal precision of the isotope measurement employing EMs is limited to shot noise (Poisson error) and independent of thermal/JN noise (Hao et al., 2022). Therefore, when the isotopic ratio is stable without drift, improving the signal statistics is key to reducing the shot noise (Poisson error) and internal presicon. Herein, we determined the relationship between the signal statistics, the measured internal precisions, and the Poisson error on the pyrite standard. The condition of ions image acquisition is described in the section Experimental. The various sizes of 10 ROIs ranged from 20 × 20 pixels^2^ (∼78 nm per pixel) to 40 × 40 pixels^2^ to 200 × 200 pixels^2^ (Supplementary Figure S1). In spot analysis, the internal precision of each spot is computed with the cycle number and the measured ratio of each cycle. In image mode, the ratio of the sum cps of all pixels ^32^S and ^34^S of a single frame is (^34^S_i_/^32^S_i_)_ROI_, and each ROI has 30 frames ratio data. The relative internal precision calculation formula is:

RSE%=100×STDS34/S32ROIMEANS34/S32ROI×130 (8)

**FIGURE 7:**
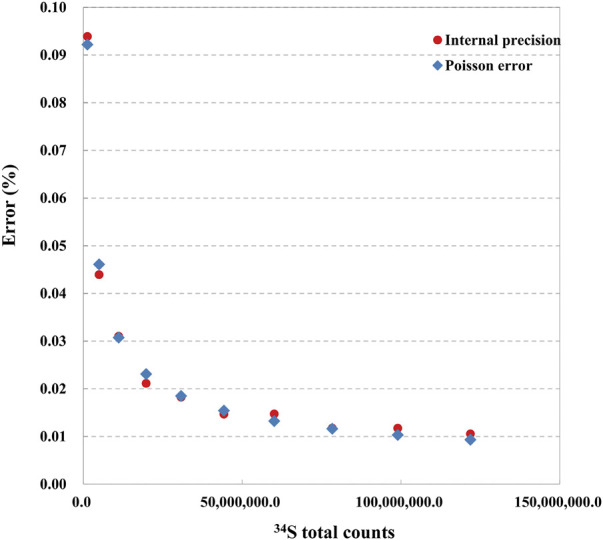
The relationship between internal precision, Poisson error, and ^34^S total counts.

In the formula, STD (^34^S_i_/^32^S_i_)_ROI_ is the standard deviation of the ratio of 30 frames in a certain ROI, and MEAN (^34^S_i_/^32^S_i_)_ROI_ is the average value of the ratio of 30 frames in a certain ROI. The Poisson error (%) is calculated following the formula in the Supplementary Note S2. As the counts of ^32^S are more than 20 times ^34^S, the statistical error of ^32^S can be ignored. The error is estimated as $\sqrt{1/N_{34S}}$ . The measured relative standard error (RSE%) and Poisson error (Poisson%) of the S isotope are shown in Figure 7, and the plot of the raw data of ^34^S/^32^S vs. the ROI with the different size used is given in Supplementary Figure S2. As shown in Figure 7, The RSE (%) and Poisson error (%) decreased exponentially relative to the ^34^S count rate and with the ROI size increasing. The internal and Poisson errors are also relatively consistent in value. In addition, the dispersion of the ratio data decreases with the ROI size increase (Supplementary Figure S2). The larger the ROI size is selected, the better internal precision and the smaller dispersion are obtained.

### 4.2 External reproducibility of ROI to ROI

#### 4.2.1 External reproducibility and signal statistics

In spot analysis, the external reproducibility is obtained from the isotopic data of spot to spot. In image mode, the external reproducibility of isotopic image analysis refers to the variation of isotopic data between ROIs. In comparison, the internal precision of the isotope image analysis refers to the dispersion of the multilayer isotope ratio within a certain ROI of the image. For the internal precision, the larger the ROI is, the more counts (signal statistics) and the higher the internal precision can be obtained, while external reproducibility is affected by more factors, such as signal statistics, sample topography, and surface potential.

**FIGURE 8:**
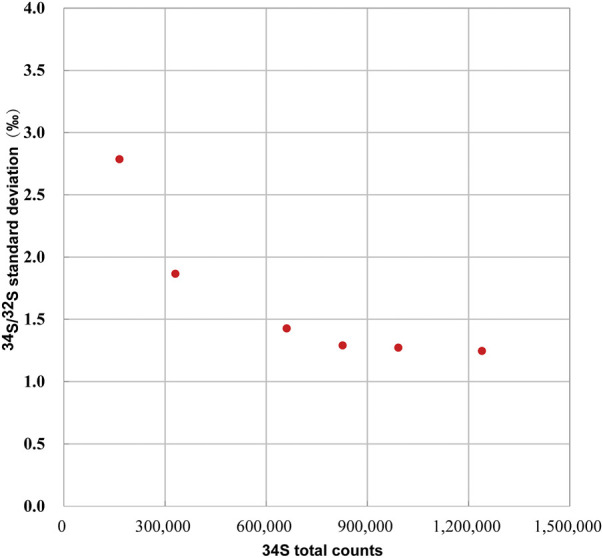
The relationship between the external reproducibility of ^34^S/^32^S and the signal statics of ^34^S total counts.

We determined the relationship between the signal statistics of ^34^S total counts and the external reproducibility of the pyrite standard of Py-1117. In the ions image with the size of 20 × 20 μm^2^ (256 × 256 piexl^2^), 100 ROIs with a size of 20 × 20 piexl^2^ (∼1.5 × 1.5 μm^2^) are randomly selected. In order to change the signal statistics of ^34^S total counts of the ROIs, different cumulative frames were adopted, which are 20, 40, 80, 100, 120, and 150 frames. The standard deviation of isotopic data of each group with the signal statics of ^34^S total counts is given in Figure 8. With the increase of statistics of ^34^S total counts, the external reproducibility of ROI to ROI is improved. However, when it is close to 1‰, the external reproducibility improvement is limited when the total counts are increased. The main reason is that the influence of signal statistics on external reproducibility can be neglected compared to other factors.

#### 4.2.2 External reproducibility and spatial resolution

**FIGURE 9:**
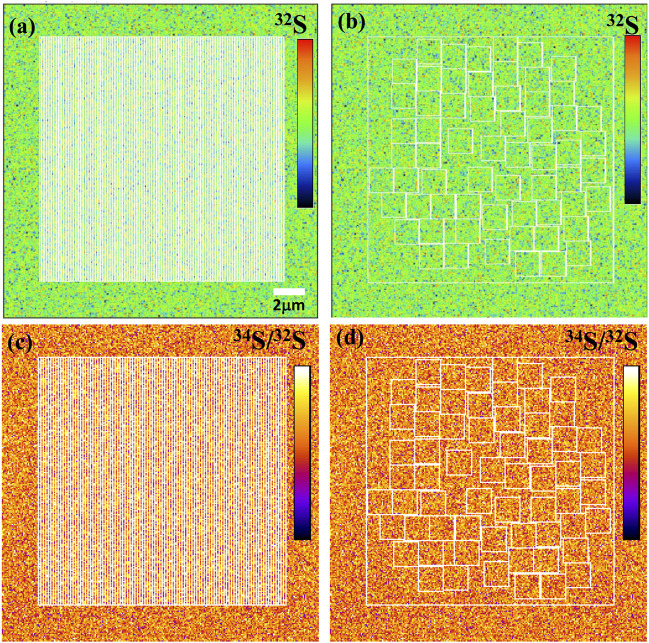
The ion image of ^32^S with the rectangular ROIs of 0.15 μm × 15 μm **(A)** and the square ROIs 1.5 μm × 1.5 μm **(B)**. The ^34^S/^32^S image with the rectangular ROIs of 0.15 μm × 15 μm **(C)** and the square ROIs 1.5 μm × 1.5 μm **(D)**.

Using ions image mode for sulfur isotope analysis, its analytical spatial resolution depends entirely on the size and the shape of ROI. The shape of ROI can be arbitrarily selected, which is the advantage of ions image analysis. For example, the square ROI with the size of 2 × 2 μm^2^ compared with the rectangular of 1 × 4 μm^2^ has the same sampling area and count statistics. However, its lateral spatial resolution has been improved from 2 μm to 1 μm. We have measured the S isotope values of ROIs with the same area size of 2.25 μm^2^ but different shapes on PY 1117 pyrite. The ROIs are shown in Figure 9, and two groups of ROIs are selected. The first group selected 100 rectangular ROIs of 0.15 μm × 15 μm in the area of 15 × 15 μm^2^ in the center of the image. For the second group, 62 square ROIs of 1.5 μm × 1.5 μm regions were randomly selected without overlapping within the same region.

**FIGURE 10:**
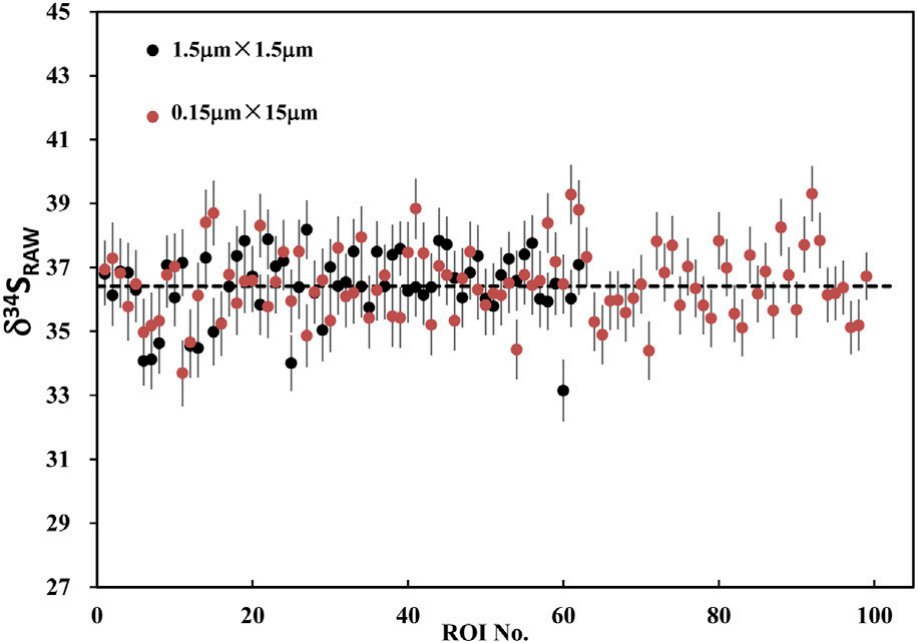
The plot of RAW δ^34^S on PY-1117 with the rectangular ROIs of 0.15 μm × 15 μm and the square ROIs of 1.5 μm × 1.5 μm.

The result of RAW δ^34^S on PY-1117 with the rectangular and square ROIs are shown in Figure 10; Supplementary Table S1. With the rectangular ROIs of 0.15 μm × 15 μm, 100 measurements yield an average RAW δ^34^S of 36.41 ± 1.1(1SD). It is 36.49 ± 1.1(1SD) with the 62 square ROIs of 1.5 μm × 1.5 μm. The results show that the different ROI shapes do not affect the isotope ratio and external reproducibility of ROI to ROI when the ROI area is the same. This result demonstrates the advantages of ions image analysis, optimizing ROI to improve spatial resolution for sample analytical requirements and ensuring higher precision by obtaining more count statistics.

### 4.2.3 The accuracy of ^34^S/^32^S measurement sample to sample

**FIGURE 11:**
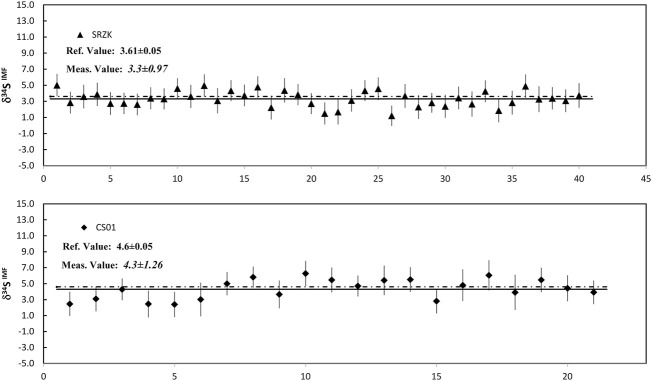
The plot of measured δ34S on PY-SRZK with the ROIs of 1.5 μm^2^ and the PY-CS01 with the ROIs of 2.5 μm^2^.

Pyrite standard Py-1117 was used as the reference material, and the sulfur isotopic measurement was conducted on the standards of PY-CS01 and PY-SRZK used as the test samples. The ^34^S/^32^S IMF measured on Py-1117 is 1.0006 with the reproducibly of 1‰ (1SD), which is used to correct the measured δ^34^S of the test samples. The corrected results of δ^34^S on PY-SRZK with the ROIs of 1.5 μm^2^ and the PY-CS01 with the ROIs of 2.5 μm^2^ are shown in Figure 11; Supplementary Table S2. The reproducibility of the S isotopic measurement on the tests sample was given in 1SD. The δ^34^S result was reported with associated 1SE uncertainty (err bar; Formula 7). To illustrate the reproducibility of the IMF between different samples, the IMF of each ROI is also shown in the table.

Forty measurements on PY-SRZK gave an average δ^34^S of 3.3 ± 1.0 (1SD), and twenty measurements on PY-CS01 gave an average δ^34^S of 4.3 ± 1.2 (1SD). The results were consistent with the recommended values of 3.6‰ ± 0.05‰ and 4.6‰ ± 0.1‰ within analytical uncertainty. The IMF was consistently shown between the two samples, with an uncertainty of 1.1‰ (1SD) obtained for the sixty measurements. In addition, the selected ROI areas were 1.5 μm^2^ and 2.5 μm^2^. Therefore, considering the primary beam spot size of ∼100 nm, the spatial resolution can reach 100 nm × 15 μm or 150 nm × 10 μm with an analytical accuracy of ∼1‰.

## 5 Conclusion

This study developed a new method that improves the spatial resolution and precision of sulfur isotopic *in-situ* analysis using NanoSIMS imaging. A focused Gaussian Cs + beam of 1 pA was utilized to achieve a lateral resolution of 100 nm for S isotopic analysis. The acquisition time of 3 h for each analytical area is applied to obtain sufficient counts. The sulfur isotopic composition was acquired by the segmentation and calculation of the secondary ion images. The deadtime, FCP, QSA, and matrix effects have been corrected to improve the analytical accuracy to ∼1‰. This method is suitable for sulfur isotopic analysis of submicron-scale to nanoscale oscillatory zoning or complex core-rim structure. More importantly, this study demonstrates that the imaging mode of NanoSIMS is a powerful tool for high spatial resolution isotope analysis, providing a strategy that can be widely applied to improve both precision and spatial resolution for the analysis of other isotopes.

## Data Availability

The original contributions presented in the study are included in the article/Supplementary Material, further inquiries can be directed to the corresponding authors.
