# Supplementary material for: NanoSIMS sulfur isotopic analysis at 100 nm scale by imaging technique
Source: Front Chem. 2023 Mar 16;11:1120092. doi: 10.3389/fchem.2023.1120092 (PMC10062601; doi:10.3389/fchem.2023.1120092)
Supplement: Supplementary file 1 [file DataSheet1.docx]

Supplementary Material for

**NanoSIMS Sulfur Isotopic Analysis at 100nm scale by Imaging Technique**

**Jia-Long Hao^1*^, Liu-Ping Zhang^2^, Wei Yang^1*^, Zhao-Yang Li^3^, Rui-Ying Li^1^, Sen Hu^1^ and Yang-Ting Lin^1^**

^1^ Key Laboratory of Earth and Planetary Physics, Institute of Geology and Geophysics, Chinese Academy of Sciences, P.O. Box 9825, Beijing 10029, China

^2^ Key Laboratory of Petroleum Resource, Institute of Geology and Geophysics, Chinese Academy of Science, Beijing 100029, China

^3^ Institute of Disaster Prevention, Sanhe 065201, China

*** Correspondence:**

Jialong Hao and Wei Yang, Key Laboratory of Earth and Planetary Physics, Institute of Geology and Geophysics, Chinese Academy of Sciences, Beijing 100029, China
sean_hao@mail.iggcas.ac.cn; yangw@mail.iggcas.ac.cn

**This file includes:**

Note S1.

Figs. S1 to S2

Tables S1 to S2

**Note S1.Deadtime correction of the electron multiplier**

Dead time effect means that the counter in the electron multiplier circuit cannot respond to multiple pulses coming from adjacent times, so the measured secondary ions intensity is lower than the actual value. The default dead time is 44 ns of EM equipped on the NanoSIMS. A linear correction model has been used to correct deadtime for most Nano SIMS experiments. Here, we take 32S ion counts as an example.

^32^S_d_=^32^S_m_ /(1- 44×10^-9^×^32^S_m_)

where ^32^S_m_ is the measured count and ^32^S_d_ is the count corrected for dead time. In the ions image mode, the dead time correction is used the same model. The total raw cps of ^32^S and ^34^S of the entire image or in the ROI area was corrected dead time before other processing.

**Note S2. Derivation of the required Ions image acquisition time**

Using NanoSIMS ions image mode, the secondary ions images can be used to calculate the elemental content or isotopic ratio of the region (ROI). According to Poisson's statistical theory, the analytical precision of 34S/32S measurements depend on total signal statistics of 34S and 32S.

The total number of ^32^S and ^34^S ions counts determines the basic analysis precession.

$Poisson(‰)=1000\times\sqrt{1/{N_{32S}}+1/{N_{34S}}}$ (1.1)

Poisson(‰) is the counting statistics error. $N_{32S}$ and $N_{34S}$ are the total counts of ^32^S and ^34^S, respectively. In the NanoSIMS image analysis, the total counts are calculated using the cumulative intensity value in the entire image or the ROI area. Indeed, the total counts depend on the primary ions beam current and the acquisition time. In order to illustrate the relationship between analytical accuracy and ions image acquisition time, the process of secondary ions generation and transmission in ions image mode has been parameterized:

The total acquisition time (t_a_) of the secondary ion image is determined by the dwell time of each pixel ( t_d_ ) and the pixel value of the image (N×N pixels). Then:

t_a_ = t_d_ × N^2^ (1.2)

Suppose N(^i^M) is the count rate of the isotope ^i^M of the element M at each pixel. It can also be understood as the counts of ^i^M secondary ions received by the electronic multiplier during the single pixel dwell time(Hoppe et al., 2013). Then:

N(^i^M)=Ip × Y × X_M_ × Ai× Yi × T (1.3)

Ip is the primary beam intensity, Y is the total sputtering ions yield, X_M_ and Ai are the content of M and the isotope abundance M, respectively. Yi is the ionization yield of isotope ^i^M, and T is the transmission efficiency of the mass spectrometer. From the formulas (1.2) and (1.3), the N_total_(^i^M) of element ^i^M in a single image is:

N_total_(^i^M)=Ip × Y × X_M_ × Ai× Yi × T × t_d_ × N^2^ (1.4)

Assuming the selected ROI is n pixel values, then:

N_ROI_(^i^M)=Ip × Y × X_M_ × Ai× Yi × T × t_d_ × n (1.5)

From the formulas (1.1) and (1.5), the counting statistics error of ROI is:

$\mathrm{Poisson}_{\mathrm{ROI}}(‰)=1000\times\sqrt{1/(Ip \times Y \times X_{M} \times Ai\times Yi \times T \times t_{d}\times n})$ (1.6)

In the isotope analysis using ions image mode, the primary beam intensity (Ip) depends on spatial resolution requirements, and the transmission efficiency (T) of the mass spectrometer is related to the used mass resolution. The content of the measuring element (X_M_), the isotope abundance(Ai), and the ions yield (Y) are determined by the experiment requirements and ion physical conditions in the matter. With the fixed values of Ip, T, and Y, the only way to improve the counting statistics would be to increase the dwell time (t_d_ ) and the ROI size (n) of the selected area. Here we estimate the required ions image acquisition time (t_a_) under the image mode instrumental condition in our method: ~1 pA primary beam with 20 × 20μm^2^ raster size and 256 × 256 pixels. In order to analyze the ^34^S/^32^S ratio on a band in the ions image with the spatial resolution of <150 nm (2 pixels), e.g., the width is 150nm (2 pixels) and 10μm (128 pixels) in length, the number of pixels with the ROI size (n) = 2 × 128 = 256. Generally, for the ^32^S of S isotope analysis, the average number K of secondary ions ejected per primary ion is ~ 0.1 (I_secondary_/I_primary_), which is K = $Y \times XM \times Ai\times Yi \times T$. According to formula 1.5, N_ROI_ (^32^S) = 0.1×1×6.24146×1000000×256× t_d_=1.6×10^8^ × td. From the formula 1.1, if the accuracy of ^34^S/^32^S is better than 1 ‰, at least 10^6^ total counts for ^34^S are required, and then the counts of ^32^S should be at least 25 ×10^6^. Therefore, the dwell time (t_d_ ) of each pixel should be larger than (25 × 10^6^ ) / (1.6 ×10^8^) = 0.16s/pixel= 160 ms/pixel. Then, according to formula 1.2, the total acquisition time (t_a_) is 0.16 ×256 × 256 ~ 10000 s. In other words, to analyze the sulfur isotope on the zoning with an area of 150nm × 10μm or other areas less than 1.5 μm^2^ and make the accuracy better than 1 ‰, the total acquisition time (t_a_) is at least more than 10000 s.

**Supplementary Figure 1.** Schematic diagram of the location and size of the square ROI selected for the internal accuracy calculation. The size of the image is 20 ×20 μm^2^.


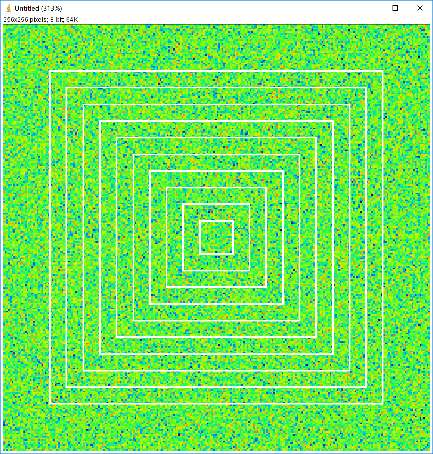


**Supplementary Figure 2.** The plot of the raw data of ^34^S/^32^S vs. the ROI with various sizes. The dispersion of the ratio data decreases with the ROI size increase. The larger the ROI size is selected, the better precision and the smaller dispersion are obtained.


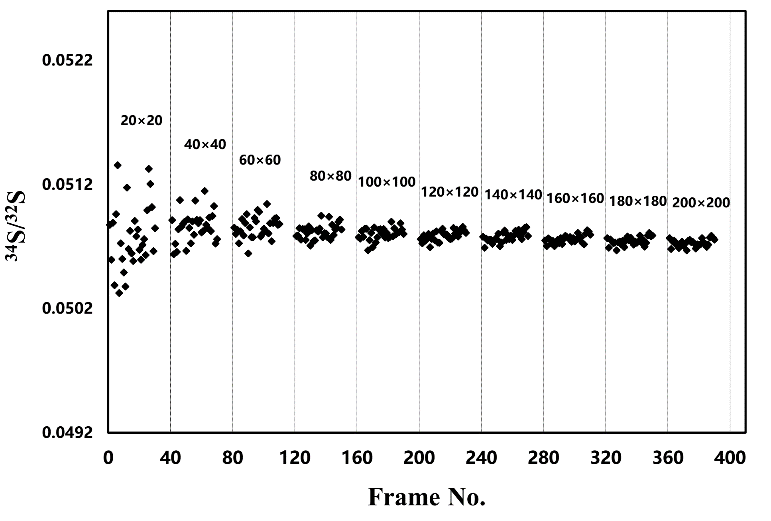


Table S1 The result of RAW δ^34^S on PY-1117 with the rectangular and square ROIs

| 0.15 μm×15μm | | |  | 1.5μm×1.5μm | | |
| --- | --- | --- | --- | --- | --- | --- |
| ^34^S/^32^S | δ^34^S_RAW_ | Err bar(1SE) |  | ^34^S/^32^S | δ^34^S_RAW_ | Err bar(1SE) |
| 0.04579 | 36.8 | 1.0 |  | 0.04579 | 36.9 | 0.9 |
| 0.04576 | 36.1 | 1.0 |  | 0.04581 | 37.3 | 1.1 |
| 0.04579 | 36.9 | 0.9 |  | 0.04579 | 36.8 | 1.1 |
| 0.04579 | 36.8 | 0.9 |  | 0.04574 | 35.8 | 1.1 |
| 0.04577 | 36.3 | 1.0 |  | 0.04577 | 36.5 | 1.1 |
| 0.04567 | 34.1 | 0.8 |  | 0.04571 | 35.0 | 1.0 |
| 0.04567 | 34.1 | 0.9 |  | 0.04572 | 35.2 | 1.0 |
| 0.04569 | 34.6 | 1.0 |  | 0.04572 | 35.3 | 1.0 |
| 0.04580 | 37.1 | 0.9 |  | 0.04579 | 36.8 | 1.0 |
| 0.04576 | 36.1 | 0.9 |  | 0.04580 | 37.0 | 1.0 |
| 0.04580 | 37.2 | 1.0 |  | 0.04565 | 33.7 | 1.0 |
| 0.04569 | 34.5 | 1.0 |  | 0.04569 | 34.7 | 1.0 |
| 0.04569 | 34.5 | 0.9 |  | 0.04576 | 36.1 | 1.0 |
| 0.04581 | 37.3 | 1.0 |  | 0.04586 | 38.4 | 1.0 |
| 0.04571 | 35.0 | 1.1 |  | 0.04587 | 38.7 | 1.0 |
| 0.04572 | 35.2 | 0.9 |  | 0.04572 | 35.2 | 1.0 |
| 0.04577 | 36.4 | 0.9 |  | 0.04579 | 36.8 | 1.0 |
| 0.04581 | 37.4 | 0.9 |  | 0.04575 | 35.9 | 1.0 |
| 0.04583 | 37.8 | 0.9 |  | 0.04578 | 36.6 | 1.0 |
| 0.04578 | 36.7 | 0.9 |  | 0.04578 | 36.6 | 1.0 |
| 0.04575 | 35.8 | 0.9 |  | 0.04585 | 38.3 | 1.0 |
| 0.04584 | 37.9 | 0.9 |  | 0.04574 | 35.8 | 1.0 |
| 0.04580 | 37.0 | 0.9 |  | 0.04578 | 36.6 | 1.0 |
| 0.04581 | 37.2 | 0.9 |  | 0.04582 | 37.5 | 1.0 |
| 0.04566 | 34.0 | 0.9 |  | 0.04575 | 35.9 | 1.0 |
| 0.04577 | 36.4 | 1.0 |  | 0.04582 | 37.5 | 1.0 |
| 0.04585 | 38.2 | 0.9 |  | 0.04570 | 34.9 | 1.0 |
| 0.04576 | 36.2 | 1.0 |  | 0.04576 | 36.2 | 1.0 |
| 0.04571 | 35.0 | 1.0 |  | 0.04578 | 36.6 | 1.0 |
| 0.04580 | 37.0 | 0.9 |  | 0.04572 | 35.3 | 1.0 |
| 0.04577 | 36.4 | 0.9 |  | 0.04582 | 37.6 | 1.0 |
| 0.04578 | 36.5 | 0.9 |  | 0.04576 | 36.1 | 1.0 |
| 0.04582 | 37.5 | 1.0 |  | 0.04576 | 36.2 | 1.0 |
| 0.04577 | 36.4 | 1.0 |  | 0.04584 | 37.9 | 1.0 |
| 0.04574 | 35.7 | 1.0 |  | 0.04573 | 35.4 | 1.0 |
| 0.04582 | 37.5 | 1.0 |  | 0.04577 | 36.3 | 1.0 |
| 0.04577 | 36.4 | 1.0 |  | 0.04579 | 36.8 | 1.0 |
| 0.04581 | 37.4 | 0.9 |  | 0.04573 | 35.5 | 1.0 |
| 0.04582 | 37.6 | 0.8 |  | 0.04573 | 35.4 | 1.0 |
| 0.04576 | 36.3 | 1.0 |  | 0.04582 | 37.5 | 1.0 |
| 0.04577 | 36.4 | 0.9 |  | 0.04588 | 38.8 | 0.9 |
| 0.04576 | 36.1 | 0.9 |  | 0.04582 | 37.4 | 0.9 |
| 0.04577 | 36.4 | 1.1 |  | 0.04572 | 35.2 | 1.0 |
| 0.04583 | 37.8 | 1.0 |  | 0.04580 | 37.1 | 0.9 |
| 0.04583 | 37.7 | 0.9 |  | 0.04579 | 36.8 | 0.9 |
| 0.04578 | 36.7 | 0.8 |  | 0.04572 | 35.3 | 0.9 |
| 0.04575 | 36.1 | 0.9 |  | 0.04578 | 36.7 | 0.9 |
| 0.04579 | 36.8 | 0.9 |  | 0.04582 | 37.5 | 0.9 |
| 0.04581 | 37.4 | 1.0 |  | 0.04577 | 36.3 | 0.9 |
| 0.04575 | 36.0 | 0.9 |  | 0.04574 | 35.8 | 0.9 |
| 0.04574 | 35.8 | 0.9 |  | 0.04576 | 36.2 | 0.9 |
| 0.04579 | 36.8 | 0.9 |  | 0.04576 | 36.1 | 0.9 |
| 0.04581 | 37.3 | 0.9 |  | 0.04578 | 36.5 | 0.9 |
| 0.04578 | 36.6 | 0.9 |  | 0.04568 | 34.4 | 0.9 |
| 0.04581 | 37.4 | 1.0 |  | 0.04579 | 36.8 | 0.9 |
| 0.04583 | 37.8 | 0.9 |  | 0.04577 | 36.4 | 0.9 |
| 0.04575 | 36.0 | 0.9 |  | 0.04578 | 36.6 | 0.9 |
| 0.04575 | 35.9 | 0.9 |  | 0.04586 | 38.4 | 0.9 |
| 0.04577 | 36.5 | 1.0 |  | 0.04580 | 37.2 | 0.9 |
| 0.04563 | 33.1 | 1.0 |  | 0.04577 | 36.5 | 0.9 |
| 0.04575 | 36.0 | 0.9 |  | 0.04590 | 39.3 | 0.9 |
| 0.04580 | 37.1 | 0.9 |  | 0.04588 | 38.8 | 0.9 |
|  |  |  |  | 0.04581 | 37.3 | 0.9 |
|  |  |  |  | 0.04572 | 35.3 | 0.9 |
|  |  |  |  | 0.04570 | 34.9 | 0.9 |
|  |  |  |  | 0.04575 | 36.0 | 0.9 |
|  |  |  |  | 0.04575 | 36.0 | 0.9 |
|  |  |  |  | 0.04573 | 35.6 | 0.9 |
|  |  |  |  | 0.04575 | 36.0 | 0.9 |
|  |  |  |  | 0.04577 | 36.5 | 0.9 |
|  |  |  |  | 0.04568 | 34.4 | 0.9 |
|  |  |  |  | 0.04583 | 37.8 | 0.9 |
|  |  |  |  | 0.04579 | 36.8 | 0.9 |
|  |  |  |  | 0.04583 | 37.7 | 0.9 |
|  |  |  |  | 0.04574 | 35.8 | 0.9 |
|  |  |  |  | 0.04580 | 37.0 | 0.9 |
|  |  |  |  | 0.04577 | 36.3 | 0.9 |
|  |  |  |  | 0.04574 | 35.8 | 0.9 |
|  |  |  |  | 0.04573 | 35.4 | 0.9 |
|  |  |  |  | 0.04583 | 37.8 | 0.9 |
|  |  |  |  | 0.04580 | 37.0 | 0.9 |
|  |  |  |  | 0.04573 | 35.6 | 0.9 |
|  |  |  |  | 0.04571 | 35.1 | 0.9 |
|  |  |  |  | 0.04581 | 37.4 | 0.9 |
|  |  |  |  | 0.04576 | 36.2 | 0.9 |
|  |  |  |  | 0.04579 | 36.9 | 0.9 |
|  |  |  |  | 0.04574 | 35.7 | 0.9 |
|  |  |  |  | 0.04585 | 38.3 | 0.9 |
|  |  |  |  | 0.04579 | 36.8 | 0.9 |
|  |  |  |  | 0.04574 | 35.7 | 0.9 |
|  |  |  |  | 0.04583 | 37.7 | 0.9 |
|  |  |  |  | 0.04590 | 39.3 | 0.9 |
|  |  |  |  | 0.04583 | 37.8 | 0.9 |
|  |  |  |  | 0.04576 | 36.1 | 0.9 |
|  |  |  |  | 0.04576 | 36.2 | 0.8 |
|  |  |  |  | 0.04577 | 36.4 | 0.8 |
|  |  |  |  | 0.04571 | 35.1 | 0.8 |
|  |  |  |  | 0.04572 | 35.2 | 0.8 |
|  |  |  |  | 0.04578 | 36.7 | 0.7 |
|  |  |  |  |  |  |  |
| *AVE.* | 36.41±1.1 | |  | *AVE.* | 36.49±1.1 | |
|  |  |  |  |  |  |  |

Table S2. The results of measured δ34S on PY-SRZK with the ROIs of 1.5μm^2^ and the PY-CS01 with the ROIs of 2.5 μm^2^.

|  |  |  |  |  |  |  |  |  |  |  |  |
| --- | --- | --- | --- | --- | --- | --- | --- | --- | --- | --- | --- |
|  | *Sample ID* | *ROI Size (μm^2^)* | *^32^S counts/pixel/frame* | *^32^S cps* | *^34^S/^32^S* | *δ^34^S_Raw_* | *K_cor_* | *δ^34^S_QSA_* | *IMF* | *δ^34^S_IMF_* | *SE^a^* |
|  | cs01-py_ROI_1 | 1.5 | 1.86E+03 | 3.72E+05 | 0.050691 | 147.8 | 0.147 | 3.0 | 0.9984 | 2.5 | 1.5 |
|  | cs01-py_ROI_2 | 1.5 | 1.86E+03 | 3.71E+05 | 0.050709 | 148.2 | 0.147 | 3.7 | 0.9991 | 3.1 | 1.6 |
|  | cs01-py_ROI_3 | 1.5 | 1.86E+03 | 3.71E+05 | 0.050759 | 149.4 | 0.147 | 4.9 | 1.0002 | 4.3 | 1.4 |
|  | cs01-py_ROI_4 | 1.5 | 1.85E+03 | 3.71E+05 | 0.050670 | 147.4 | 0.147 | 3.0 | 0.9984 | 2.5 | 1.7 |
|  | cs01-py_ROI_5 | 1.5 | 1.85E+03 | 3.71E+05 | 0.050667 | 147.3 | 0.147 | 3.0 | 0.9984 | 2.4 | 1.6 |
|  | cs01-py_ROI_6 | 1.5 | 1.85E+03 | 3.70E+05 | 0.050686 | 147.7 | 0.147 | 3.6 | 0.9990 | 3.0 | 2.1 |
|  | cs01-py_ROI_7 | 1.5 | 1.85E+03 | 3.70E+05 | 0.050770 | 149.6 | 0.146 | 5.6 | 1.0009 | 5.0 | 1.5 |
|  | cs01-py_ROI_8 | 1.5 | 1.85E+03 | 3.70E+05 | 0.050804 | 150.4 | 0.146 | 6.4 | 1.0018 | 5.8 | 1.3 |
|  | cs01-py_ROI_9 | 1.5 | 1.85E+03 | 3.70E+05 | 0.050706 | 148.2 | 0.146 | 4.2 | 0.9996 | 3.7 | 1.7 |
|  | cs01-py_ROI_10 | 1.5 | 1.85E+03 | 3.70E+05 | 0.050818 | 150.7 | 0.146 | 6.8 | 1.0022 | 6.3 | 1.6 |
|  | cs01-py_ROI_11 | 1.5 | 1.85E+03 | 3.69E+05 | 0.050781 | 149.9 | 0.146 | 6.0 | 1.0014 | 5.5 | 1.5 |
|  | cs01-py_ROI_12 | 1.5 | 1.85E+03 | 3.69E+05 | 0.050742 | 149.0 | 0.146 | 5.3 | 1.0007 | 4.7 | 1.3 |
|  | cs01-py_ROI_13 | 1.5 | 1.85E+03 | 3.69E+05 | 0.050771 | 149.6 | 0.146 | 6.0 | 1.0014 | 5.4 | 1.8 |
|  | cs01-py_ROI_14 | 1.5 | 1.85E+03 | 3.69E+05 | 0.050775 | 149.7 | 0.146 | 6.1 | 1.0015 | 5.5 | 1.5 |
|  | cs01-py_ROI_15 | 1.5 | 1.84E+03 | 3.69E+05 | 0.050655 | 147.0 | 0.146 | 3.4 | 0.9988 | 2.8 | 1.5 |
|  | cs01-py_ROI_16 | 1.5 | 1.84E+03 | 3.69E+05 | 0.050741 | 149.0 | 0.146 | 5.4 | 1.0008 | 4.8 | 2.0 |
|  | cs01-py_ROI_17 | 1.5 | 1.84E+03 | 3.68E+05 | 0.050784 | 149.9 | 0.146 | 6.6 | 1.0020 | 6.0 | 1.9 |
|  | cs01-py_ROI_18 | 1.5 | 1.84E+03 | 3.68E+05 | 0.050684 | 147.7 | 0.146 | 4.5 | 0.9999 | 3.9 | 2.2 |
|  | cs01-py_ROI_19 | 1.5 | 1.84E+03 | 3.68E+05 | 0.050746 | 149.1 | 0.145 | 6.0 | 1.0014 | 5.5 | 1.5 |
|  | cs01-py_ROI_20 | 1.5 | 1.84E+03 | 3.67E+05 | 0.050694 | 147.9 | 0.145 | 5.0 | 1.0004 | 4.4 | 1.6 |
|  | cs01-py_ROI_21 | 1.5 | 1.84E+03 | 3.67E+05 | 0.050671 | 147.4 | 0.145 | 4.5 | 0.9999 | 3.9 | 1.5 |
| *Average* |  |  |  |  |  |  |  |  |  | *4.3* |  |
| *STD* |  |  |  |  |  |  |  |  |  | *1.2^b^* |  |
|  | SRZK-py_ROI_1 | 2.5 | 1.83E+03 | 3.66E+05 | 0.050789 | 150.0 | 0.147 | 5.6 | 1.0020 | 5.0 | 1.4 |
|  | SRZK-py_ROI_2 | 2.5 | 1.87E+03 | 3.75E+05 | 0.050857 | 151.6 | 0.151 | 3.4 | 0.9998 | 2.8 | 1.3 |
|  | SRZK-py_ROI_3 | 2.5 | 1.87E+03 | 3.74E+05 | 0.050884 | 152.2 | 0.150 | 4.2 | 1.0006 | 3.6 | 1.5 |
|  | SRZK-py_ROI_4 | 2.5 | 1.87E+03 | 3.74E+05 | 0.050889 | 152.3 | 0.150 | 4.4 | 1.0008 | 3.9 | 1.5 |
|  | SRZK-py_ROI_5 | 2.5 | 1.87E+03 | 3.73E+05 | 0.050828 | 150.9 | 0.150 | 3.3 | 0.9997 | 2.7 | 1.4 |
|  | SRZK-py_ROI_6 | 2.5 | 1.87E+03 | 3.73E+05 | 0.050828 | 150.9 | 0.150 | 3.3 | 0.9997 | 2.7 | 1.3 |
|  | SRZK-py_ROI_7 | 2.5 | 1.87E+03 | 3.73E+05 | 0.050820 | 150.7 | 0.150 | 3.2 | 0.9996 | 2.6 | 1.3 |
|  | SRZK-py_ROI_8 | 2.5 | 1.86E+03 | 3.73E+05 | 0.050848 | 151.4 | 0.150 | 4.0 | 1.0004 | 3.4 | 1.4 |
|  | SRZK-py_ROI_9 | 2.5 | 1.86E+03 | 3.73E+05 | 0.050842 | 151.2 | 0.150 | 3.9 | 1.0003 | 3.3 | 1.3 |
|  | SRZK-py_ROI_10 | 2.5 | 1.83E+03 | 3.67E+05 | 0.050782 | 149.9 | 0.147 | 5.2 | 1.0016 | 4.6 | 1.3 |
|  | SRZK-py_ROI_11 | 2.5 | 1.86E+03 | 3.72E+05 | 0.050849 | 151.4 | 0.150 | 4.2 | 1.0006 | 3.6 | 1.4 |
|  | SRZK-py_ROI_12 | 2.5 | 1.84E+03 | 3.67E+05 | 0.050807 | 150.5 | 0.147 | 5.5 | 1.0019 | 5.0 | 1.4 |
|  | SRZK-py_ROI_13 | 2.5 | 1.84E+03 | 3.67E+05 | 0.050728 | 148.7 | 0.147 | 3.6 | 1.0000 | 3.1 | 1.6 |
|  | SRZK-py_ROI_14 | 2.5 | 1.86E+03 | 3.72E+05 | 0.050874 | 152.0 | 0.150 | 4.9 | 1.0013 | 4.3 | 1.3 |
|  | SRZK-py_ROI_15 | 2.5 | 1.86E+03 | 3.72E+05 | 0.050846 | 151.3 | 0.149 | 4.3 | 1.0007 | 3.7 | 1.4 |
|  | SRZK-py_ROI_16 | 2.5 | 1.86E+03 | 3.72E+05 | 0.050887 | 152.3 | 0.149 | 5.3 | 1.0017 | 4.8 | 1.3 |
|  | SRZK-py_ROI_17 | 2.5 | 1.86E+03 | 3.72E+05 | 0.050772 | 149.7 | 0.149 | 2.8 | 0.9992 | 2.2 | 1.5 |
|  | SRZK-py_ROI_18 | 2.5 | 1.86E+03 | 3.71E+05 | 0.050863 | 151.7 | 0.149 | 4.9 | 1.0013 | 4.4 | 1.5 |
|  | SRZK-py_ROI_19 | 2.5 | 1.86E+03 | 3.71E+05 | 0.050837 | 151.1 | 0.149 | 4.4 | 1.0008 | 3.8 | 1.3 |
|  | SRZK-py_ROI_20 | 2.5 | 1.85E+03 | 3.71E+05 | 0.050781 | 149.9 | 0.149 | 3.3 | 0.9997 | 2.7 | 1.3 |
|  | SRZK-py_ROI_21 | 2.5 | 1.85E+03 | 3.71E+05 | 0.050726 | 148.6 | 0.149 | 2.1 | 0.9985 | 1.5 | 1.4 |
|  | SRZK-py_ROI_22 | 2.5 | 1.85E+03 | 3.70E+05 | 0.050724 | 148.6 | 0.149 | 2.2 | 0.9986 | 1.7 | 1.6 |
|  | SRZK-py_ROI_23 | 2.5 | 1.85E+03 | 3.70E+05 | 0.050786 | 150.0 | 0.149 | 3.7 | 1.0001 | 3.1 | 1.4 |
|  | SRZK-py_ROI_24 | 2.5 | 1.85E+03 | 3.70E+05 | 0.050840 | 151.2 | 0.149 | 4.9 | 1.0013 | 4.3 | 1.3 |
|  | SRZK-py_ROI_25 | 2.5 | 1.85E+03 | 3.70E+05 | 0.050849 | 151.4 | 0.149 | 5.1 | 1.0015 | 4.6 | 1.4 |
|  | SRZK-py_ROI_26 | 2.5 | 1.85E+03 | 3.70E+05 | 0.050700 | 148.0 | 0.149 | 1.8 | 0.9982 | 1.2 | 1.3 |
|  | SRZK-py_ROI_27 | 2.5 | 1.85E+03 | 3.70E+05 | 0.050807 | 150.4 | 0.149 | 4.2 | 1.0006 | 3.7 | 1.5 |
|  | SRZK-py_ROI_28 | 2.5 | 1.85E+03 | 3.70E+05 | 0.050743 | 149.0 | 0.149 | 2.9 | 0.9993 | 2.3 | 1.5 |
|  | SRZK-py_ROI_29 | 2.5 | 1.85E+03 | 3.70E+05 | 0.050759 | 149.4 | 0.148 | 3.4 | 0.9998 | 2.8 | 1.2 |
|  | SRZK-py_ROI_30 | 2.5 | 1.84E+03 | 3.68E+05 | 0.050704 | 148.1 | 0.148 | 2.9 | 0.9993 | 2.4 | 1.4 |
|  | SRZK-py_ROI_31 | 2.5 | 1.85E+03 | 3.69E+05 | 0.050782 | 149.9 | 0.148 | 4.0 | 1.0004 | 3.4 | 1.4 |
|  | SRZK-py_ROI_32 | 2.5 | 1.85E+03 | 3.69E+05 | 0.050747 | 149.1 | 0.148 | 3.2 | 0.9996 | 2.7 | 1.6 |
|  | SRZK-py_ROI_33 | 2.5 | 1.85E+03 | 3.69E+05 | 0.050812 | 150.6 | 0.148 | 4.8 | 1.0012 | 4.2 | 1.3 |
|  | SRZK-py_ROI_34 | 2.5 | 1.84E+03 | 3.69E+05 | 0.050705 | 148.1 | 0.148 | 2.4 | 0.9988 | 1.9 | 1.5 |
|  | SRZK-py_ROI_35 | 2.5 | 1.84E+03 | 3.69E+05 | 0.050746 | 149.1 | 0.148 | 3.4 | 0.9998 | 2.8 | 1.5 |
|  | SRZK-py_ROI_36 | 2.5 | 1.84E+03 | 3.69E+05 | 0.050834 | 151.1 | 0.148 | 5.5 | 1.0018 | 4.9 | 1.4 |
|  | SRZK-py_ROI_37 | 2.5 | 1.84E+03 | 3.68E+05 | 0.050755 | 149.3 | 0.148 | 3.8 | 1.0002 | 3.3 | 1.6 |
|  | SRZK-py_ROI_38 | 2.5 | 1.84E+03 | 3.68E+05 | 0.050757 | 149.3 | 0.148 | 3.9 | 1.0003 | 3.4 | 1.4 |
|  | SRZK-py_ROI_39 | 2.5 | 1.84E+03 | 3.68E+05 | 0.050742 | 149.0 | 0.148 | 3.6 | 1.0000 | 3.1 | 1.4 |
|  | SRZK-py_ROI_40 | 2.5 | 1.84E+03 | 3.68E+05 | 0.050763 | 149.5 | 0.148 | 4.3 | 1.0007 | 3.7 | 1.5 |
| *Average* |  |  |  |  |  |  |  |  |  | *3.3* |  |
| *STD* |  |  |  |  |  |  |  |  | 1.1^c^ | *1.0* |  |
|  |  |  |  |  |  |  |  |  |  |  |  |

1. The δ^34^S result was reported with associated 1SE uncertainty (err bar), which is estimated as the square sum of the reproducibility of δ^34^S measurements on the corresponding reference pyrite of PY 1117 (SD_standard_), the internal precision of each ROI on the sample (SE_sample_) and the uncertainty of the reference values of the standards 1117 (SD).
2. The reproducibility of the S isotopic measurement was given 1SD.
3. The reproducibility IMF of all measuremtns on the two samples of CS01 and SRZK was given in 1SD.
